# Oxidative stress in acute pancreatitis induced acute lung injury: mechanisms and therapeutic advances

**DOI:** 10.7717/peerj.20905

**Published:** 2026-03-27

**Authors:** Yang Jun, Chen Jianmei, Liu Jing, Yang Guixiang, Guo Jinwei, Li Jianhua

**Affiliations:** 1 Department of Critical Care Medicine, Chongqing University Jiangjin Hospital, Chongqing, China; 2 Department of Trauma Surgery, Chongqing University Jiangjin Hospital, Chongqing, China; 3 Department of Critical Care Medicine, The First Affiliated Hospital of Gannan Medical University, Ganzhou, Jiangxi Province, China

**Keywords:** Acute pancreatitis, Lung injury, Oxidative stress, Reactive oxygen species (ROS), Therapeutic advances

## Abstract

Acute pancreatitis (AP) is a common acute abdominal condition. The trigger of acute lung injury (ALI) is a critical factor affecting unfavorable patient outcomes. Evidence indicates that the mechanisms underlying AP-ALI involve a complex bidirectional interaction between oxidative stress and inflammatory processes. Specifically, the overproduction of reactive oxygen species (ROS), combined with impairment of the antioxidant defense system, is a key aspect of AP-ALI pathophysiology. These biochemical changes, in turn, promote the secretion of inflammatory mediators through the activation of key signaling pathways, such as NF-κB and MAPK, which ultimately lead to damage to lung tissue. Additionally, this process involves changes at the molecular level, including mitochondrial dysfunction, endoplasmic reticulum stress, and programmed cell death. Although preclinical studies in animal models suggest that antioxidant therapy may offer protective effects, applying such treatments in clinical settings faces significant hurdles, including pharmacokinetic issues and side effects. This article provides a comprehensive review of the role of oxidative stress in the development and progression of AP-ALI, examining the potential for creating innovative therapies based on these mechanisms.

## Introduction

**Figure 1:**
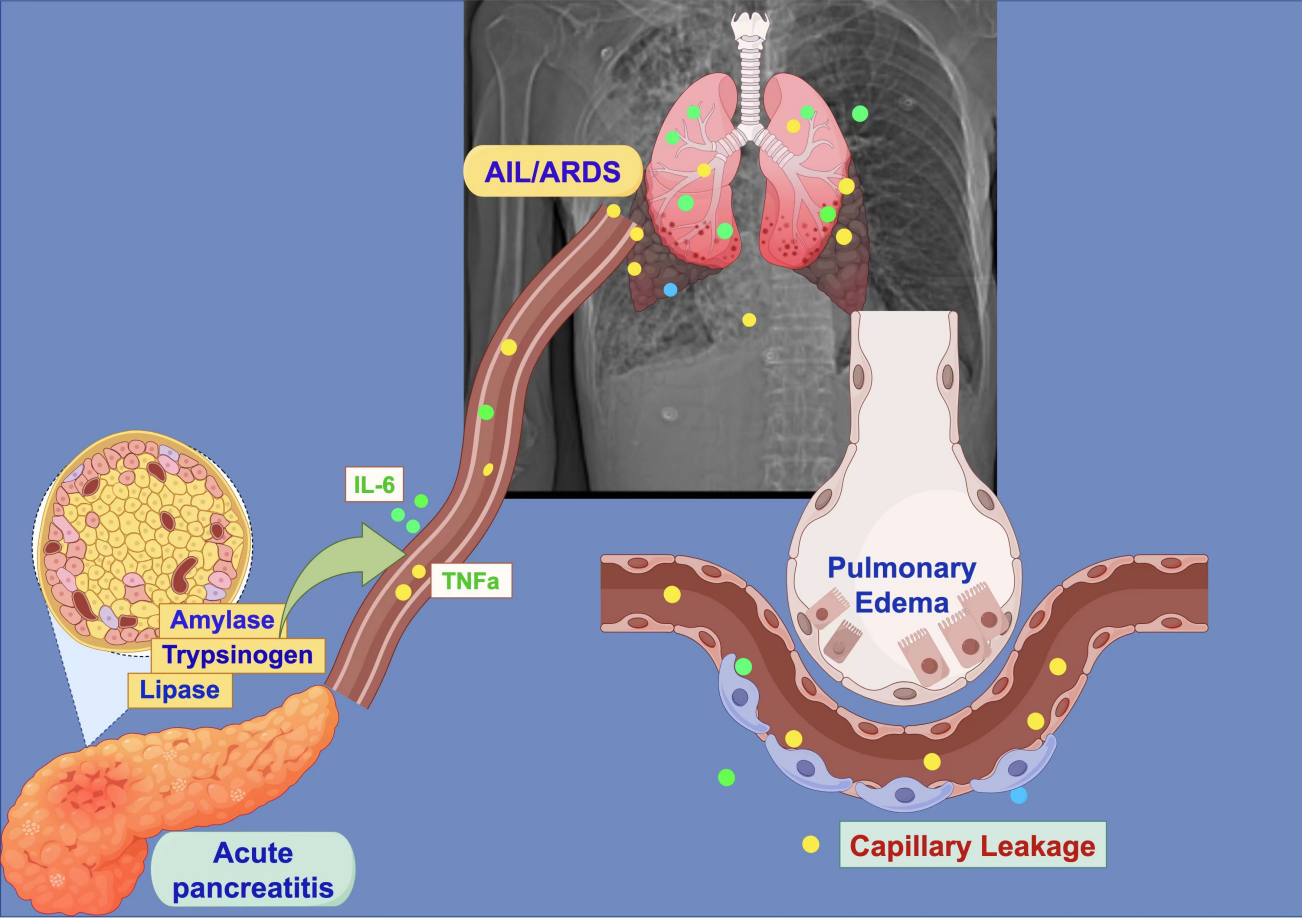
Schematic diagram of pancreatitis-induced acute lung injury/acute respiratory distress syndrome (ALI/ARDS). Created with Figdraw 2.0: https://www.figdraw.com.

Acute pancreatitis (AP) is an inflammatory condition characterized by the autodigestion of pancreatic tissues (Lankisch, Apte & Banks, 2015), with its complication, acute lung injury (ALI), significantly affecting patient outcomes (Samanta et al., 2018). Recent studies suggest that oxidative stress plays a crucial role in the development of AP-ALI (Cai, Yang & Huang, 2024), primarily due to an imbalance between the production of reactive oxygen species (ROS) and their clearance mechanisms. In the early stages of the disease, the abnormal activation of digestive zymogens in pancreatic acinar cells triggers oxidative stress, leading to mitochondrial dysfunction and excessive ROS production. These oxidative byproducts enter the bloodstream and reach the lungs. There, they activate transcription factors, such as NF-κB, which in turn increase the expression of inflammatory mediators like TNF-α and IL-6 (Chen et al., 2016). This process ultimately damages the alveolar-capillary barrier (Ge et al., 2020; Sarr et al., 2013) (As shown in Fig. 1).

The interaction between oxidative stress and inflammatory responses sustains a harmful cycle in AP-ALI. Studies using animal models of chemically induced AP have shown notable increases in the oxidative damage marker malondialdehyde (MDA) in the pancreas and significant decreases in superoxide dismutase (SOD) activity (Hagar et al., 2020). Additionally, lung tissues show infiltration of inflammatory cells and interstitial edema, linking pancreatic oxidative damage to lung inflammation. Mechanistic studies suggest that endoplasmic reticulum stress impacts the HO-1/HIF-1α signaling pathway. This, in turn, influences the expression of the Golgi protein GM130, leading to cell death caused by oxidative stress.

Current therapeutic approaches targeting oxidative stress have become a main focus in AP-ALI research (Chen et al., 2024). Experimental investigations have revealed that thiol compounds, such as N-acetylcysteine, offer protective benefits by maintaining glutathione levels (Minati et al., 2021). Similarly, plant-derived compounds like resveratrol have been shown to inhibit NADPH oxidase (NOX) activity by modulating the Sirt1/AMPK pathway (Niu, Zhang & Okolo 3rd, 2025). Additionally, the epigenetic regulation of the miR-122-5p/DUSP4 axis and the bioactive peptide Hydrostatin-SN10 have proven effective in disrupting the oxidative-inflammatory network (Pădureanu et al., 2022). These findings provide a foundation for developing targeted therapeutic interventions, although more research is needed to assess the safety and effectiveness of antioxidant treatments.

We think that the excessive production of ROS combined with dysfunction of the antioxidant defense system represents a key pathway in the pathophysiology of AP-ALI, making it crucial for developing targeted therapies. Therefore, this article provides a comprehensive review of the role of oxidative stress in the pathogenesis and progression of AP-ALI, and explores the potential for developing novel therapies based on these mechanisms. It offers the latest insights for gastroenterologists, pulmonologists, basic medical researchers, and clinicians focused on comorbidities management, aiming to advance targeted therapy development and optimize clinical management strategies.

## Survey Methodology

Given that this article aims to provide a comprehensive overview of the potential mechanisms and therapeutic advances of the interaction between AP-ALI and oxidative stress—rather than conducting a rigorous quantitative analysis—we adopted a narrative review approach to synthesize the literature. The literature search covered PubMed, Web of Science, and the China National Knowledge Infrastructure (CNKI) database. The search encompassed articles published within the past five years and earlier relevant publications. Search keywords included: acute pancreatitis and acute lung injury, inflammatory cascade in acute pancreatitis, pathological mechanisms of acute lung injury, oxidative stress and lung injury, oxidative stress and inflammation, oxidative stress and programmed cell death, oxidative stress and alveolar barrier damage. As research progressed, the search strategy was progressively optimized, incorporating additional keywords such as: NF-κB signaling pathway and AP-ALI, Nrf2/ARE pathway and AP-ALI, MAPK and PI3K/Akt signaling pathways and AP-ALI, oxidative stress markers and AP-ALI, and intervention strategies targeting antioxidant mechanisms. Following removal of duplicates and irrelevant articles, 63 articles were ultimately included in this review.

## Molecular Mechanisms of Pancreatitis-Induced Acute Lung Injury (As shown in Fig. 2)

### Pathophysiological linkage mechanisms between acute pancreatitis and acute lung injury

#### Pathological impact of pancreatitis inflammatory cascade on pulmonary tissues

The cause of AP mainly involves systemic inflammatory responses triggered by injury to pancreatic acinar cells (Sastre et al., 2025). Damaged acinar cells release digestive enzymes such as lipase and amylase, along with the inactive precursor trypsinogen, which then activate the NF-κB signaling pathway. This activation results in the release of pro-inflammatory cytokines, including TNF-α and IL-6. These cytokines then spread through the systemic circulation to the lungs, causing dysfunction of the pulmonary vascular endothelial barrier (Mihoc et al., 2024; Yu & Kim, 2014). Empirical evidence shows a significant increase in oxidative stress markers (*e.g.*, MDA, NOX) along with a decrease in antioxidants (*e.g.*, GSH, TAC) within the lung tissues of animal models of AP, highlighting the critical role of oxidative stress in lung injury associated with pancreatitis.

**Figure 2:**
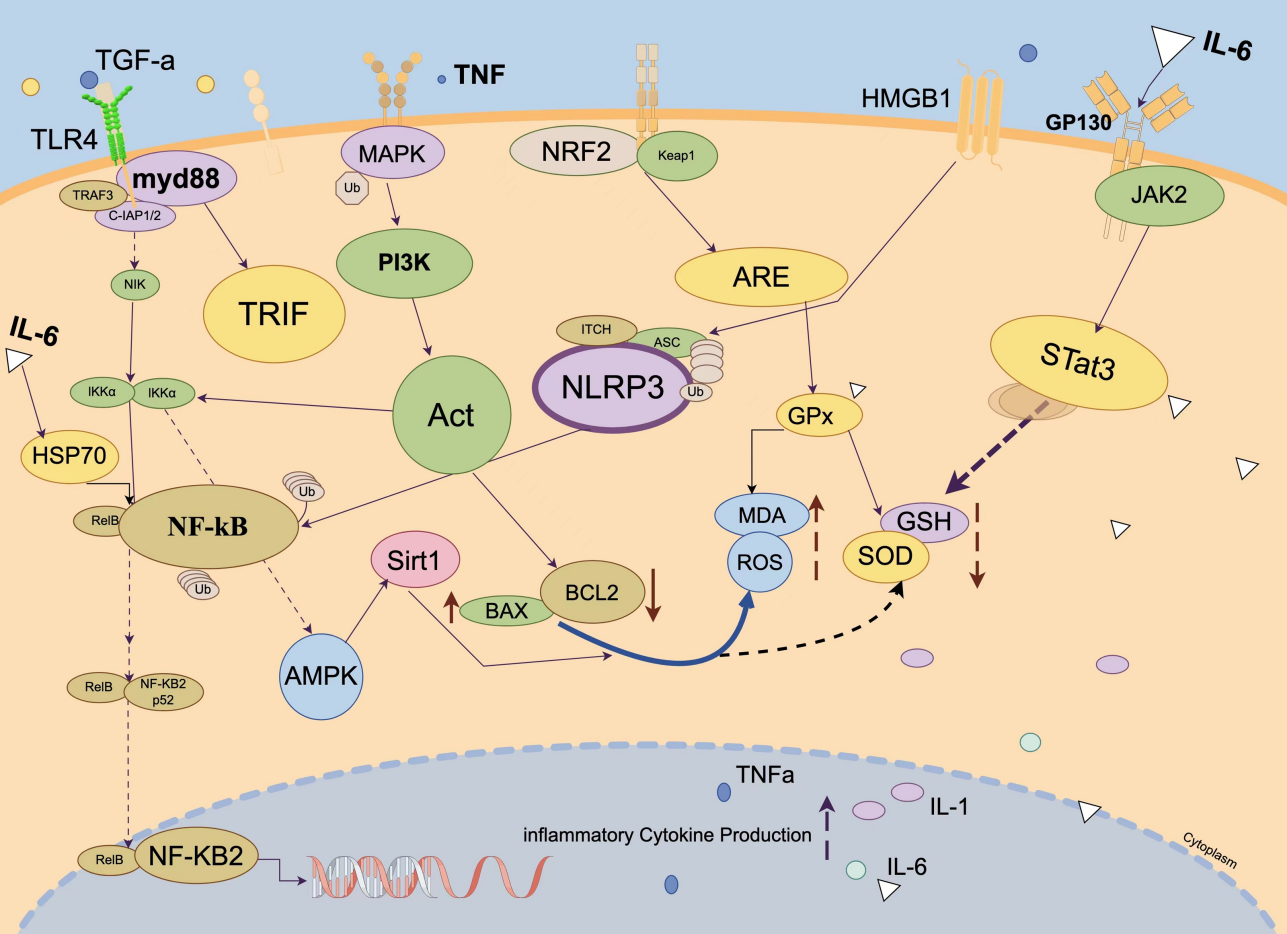
Molecular mechanisms of pancreatitis-induced acute lung injury. Created with Figdraw 2.0: https://www.figdraw.com.

#### Pathological alterations in ali and their association with pancreatitis

ALI, a common and severe complication in patients with acute pancreatitis (AP), is mainly characterized by the disruption of the alveolar-capillary membrane’s integrity, pulmonary interstitial edema, and infiltration of neutrophils (Alabdul Razzak et al., 2025; Kandikattu et al., 2023). The mechanisms behind its development are complex: Pancreas-derived inflammatory mediator HMGB1 activates pulmonary macrophages through the TLR4/MyD88/TRIF signaling pathway, leading to the release of cytokines such as TNF-α and IL-1β. At the same time, oxidative stress reduces superoxide dismutase (SOD) activity in lung tissues, worsening cellular damage (Ge et al., 2020; Liu et al., 2005; Correia de Sá & Rocha, 2023), and ultimately causing cell death. Animal studies show notable changes in respiratory parameters (*e.g.*, PIF, FVC) in rat models with AP. Flavonoids like baicalin help reduce these changes by blocking the TLR4/NF-κB signaling pathway. Additionally, systemic inflammation caused by AP promotes the recruitment of pulmonary neutrophils *via* the P2X7 receptor and LAMC2-neutrophil pathways, worsening tissue damage (Chen et al., 2025). Importantly, the Tibetan herbal formula Sichen significantly lowers inflammatory cell counts in bronchoalveolar lavage fluid by modulating TLR4 signaling, opening new avenues for AP-ALI treatment research (Yan et al., 2022).

#### The relationship between intestinal inflammation, acute pancreatitis, and acute lung injury

Acute pancreatitis triggers the accumulation and infiltration of inflammatory cells and cytokines, which disrupt the intestinal barrier and mucosal structure. This damage increases intestinal mucosal permeability and impairs the intestine’s mechanical, chemical, immunological, and biological defenses, alongside mesenteric lymphatic dysfunction (Ge et al., 2020). Consequently, gut microbiota dysbiosis occurs, allowing bacteria, endotoxins, and associated toxic factors to translocate into systemic circulation and reach the lungs *via* lymphatic and hematogenous routes.In the lungs, inflammatory activators such as endotoxins, HMGB1, and pro-inflammatory cytokines activate signaling pathways like NF-κB, leading to the release of a cascade of inflammatory mediators, including TNF-α, IL-1β, and IL-6 (Zhang et al., 2016). These mediators directly injure the pulmonary microvascular endothelium and alveolar epithelium, increasing vascular permeability. They also further activate and recruit pulmonary neutrophils, promoting neutrophil extracellular traps (NETs) release and oxidative stress, thereby exacerbating acute lung injury. Concurrently, oxidative stress during AP creates an imbalance between oxidation and antioxidant defense. Neutrophilic infiltration and increased protease secretion generate oxidative intermediates, such as oxygen free radicals and lipid peroxides, contributing to early intestinal epithelial damage. Furthermore, in the injured intestinal tissue, activation of the Nrf2/ARE antioxidant signaling pathway may be insufficient to compensate, potentially leading to reduced levels of key antioxidants like total glutathione (GSH) and superoxide dismutase (SOD) (Liu et al., 2022). This impairment in antioxidant capacity exacerbates intestinal barrier dysfunction, disrupts mucosal architecture, and perpetuates increased intestinal permeability.

### Molecular regulatory mechanisms of oxidative stress in AP-ALI

#### ROS generation and its pulmonary injury effects

The overproduction of reactive oxygen species (ROS) is a key factor contributing to oxidative stress in the development of ALI related to acute pancreatitis (AP) (Xia et al., 2024). The main sources of ROS include nicotinamide adenine dinucleotide phosphate (NADPH) oxidase (NOX) systems and the mitochondrial electron transport chain. Additionally, the infiltration of inflammatory cells, specifically neutrophils and macrophages, further increases ROS production (Segal & Abo, 1993). Experimental studies have shown significantly higher levels of oxidative stress biomarkers, such as malondialdehyde (MDA), along with increased activity of NOX enzymes, and a decrease in the activity of antioxidant defenses, including SOD and glutathione (GSH), are observed within the lung tissues of AP-ALI models. Therefore, this indicates a disruption in the balance between oxidative and antioxidant processes (Fu et al., 2018). ROS are known to induce oxidative modifications in biomacromolecules, including lipids, proteins, and DNA. This leads to the compromise of the structural integrity of pulmonary epithelial cells and the activation of pro-inflammatory signaling pathways, such as nuclear factor-kappa B (NF-κB), which exacerbate damage to pulmonary tissue. In models of AP-ALI induced by sodium taurocholate, the accumulation of ROS has been positively correlated with the degree of alveolar edema and infiltration of inflammatory cells.

#### Interplay between oxidative stress and inflammatory responses

In the pathology of acute pancreatitis-associated lung injury (AP-ALI), oxidative stress and inflammation form a synergistic feedback loop. ROS play a dual role by activating both the NF-κB transcription factor and the NLRP3 inflammasome complex; the latter, in turn, triggers the release of pro-inflammatory cytokines such as TNF-α and IL-6. These cytokines not only promote neutrophil infiltration but also increase the expression of NADPH oxidases (NOX), leading to more ROS production (Amiti Tamizhselvi & Manickam, 2019). Supporting this, data from murine models of AP-ALI induced by L-arginine reveal a significant positive correlation between the phosphorylation levels of NF-κB in the lungs and the concentrations of IL-6 and TNF-α (Jin et al., 2019). Additionally, inflammatory mediators like HMGB1 (Park et al., 2018) have been shown to weaken cellular antioxidant defenses by inhibiting the activation of the Nrf2 signaling pathway. Furthermore, the activation of the TLR4/MyD88/TRIF signaling cascade has been identified as a key molecular link connecting oxidative stress with inflammatory responses.

#### Oxidative stress-induced programmed cell death and alveolar barrier injury

Oxidative stress is known to damage the integrity of the alveolar-capillary barrier and induce apoptosis in pulmonary epithelial cells through various mechanisms. In models of ALI, ROS significantly increases the levels of pro-apoptotic proteins such as BAX and Caspase-3. Simultaneously, they suppress the activity of the anti-apoptotic protein Bcl-2 *via* mitochondrial apoptotic pathways and endoplasmic reticulum stress (Xia et al., 2019). Pharmacological studies have demonstrated that cholinesterase inhibitors, such as rivastigmine, can significantly reduce pulmonary cell apoptosis by modulating the HSP70/Caspase-3 signaling pathway. Notably, oxidative stress causes oxidative modifications and subsequent degradation of tight junction proteins, including ZO-1 and occludin, which heighten the permeability of alveolar epithelial cells, leading to protein exudation and pulmonary edema (Chen, Xie & Wu, 2023). Additionally, recent research focusing on the Spef2/AMPKα/Sirt1 signaling pathway indicates that its activation significantly alleviates oxidative stress-induced apoptosis and barrier dysfunction, revealing new molecular targets for possible therapeutic interventions.

#### NETs-induced oxidative stress pathway

Early studies revealed that during AP, large numbers of neutrophils rapidly recruit from the peripheral circulation to the site of pancreatic inflammation, where neutrophil-generated oxygen free radicals cause pulmonary endothelial cell injury (Guice et al., 1989). In AP-ALI, a vicious cycle exists between neutrophil extracellular trap (NET) formation and oxidative stress. During AP onset, pancreatic-released DAMPs, IL-8, and complement activation products (C5a) activate neutrophils (Pan & Lee, 2024). The key mechanism involves activating NADPH oxidase (NOX), generating massive superoxide anions (O_2_•^−^) that convert to hydrogen peroxide (H_2_O_2_) (Wan et al., 2020). These ROS not only promote chromatin depolymerization and NET release by activating myeloperoxidase (MPO), neutrophil elastase (NE), and peptidyl arginine deiminase 4 (PAD4), but also act as toxic molecules attacking the alveolar-capillary barrier. Furthermore, NETs serve as potent stimulators of Mac-1 expression and ROS formation in isolated neutrophils. Thus, NET release signifies elevated local oxidative stress levels. MPO within NET structures utilizes H_2_O_2_ generated during these processes to catalyze the formation of potent oxidants like hypochlorous acid (HOCI) (Scozzi et al., 2022), causing direct damage to membrane lipids and proteins in alveolar epithelial and endothelial cells. This provides a theoretical basis for therapeutic strategies targeting key steps in NET formation—such as using PAD4 inhibitors, DNase I to degrade the NET scaffold, or MPO inhibitors and antioxidants—thereby improving patient outcomes.

### Regulatory networks of oxidative stress-associated signaling pathways in AP-ALI

#### Pathophysiological role of the NF-κB signaling pathway

The NF-κB signaling pathway plays a crucial regulatory role in the development of acute pancreatitis-associated lung injury (AP-ALI) (Tak & Firestein, 2001). Basic research indicates that necrosis in pancreatic acinar cells during the early stages of acute pancreatitis is closely associated with inflammatory responses mediated by NF-κB (Geng et al., 2024; Hu et al., 2022; Zhu et al., 2018). Studies into the mechanisms involved indicate that the AMPKα/Sirt1 signaling pathway inhibits the transcriptional activity of NF-κB through deacetylation. This inhibition reduces inflammatory damage in both pancreatic and lung tissues (Zhao et al., 2017). Importantly, the Spef2 protein, a newly identified regulatory molecule, indirectly reduces the pro-inflammatory effects of NF-κB by activating the AMPKα/Sirt1 pathway (Jin et al., 2017). Additionally, pharmacological studies demonstrate that Riva-a, a drug whose specific nature requires clarification, significantly lowers levels of lung inflammatory cytokines, MDA, and NOx by blocking the HSP70/IL-6/NF-κB signaling pathway (Yehia Kamel et al., 2023). These results provide a scientific foundation for creating targeted treatments for AP-ALI.

#### Dysfunction and modulation of the Nrf2/ARE antioxidant system

The Nrf2/ARE signaling pathway is a key part of cellular antioxidant defense and is notably impaired ALI (Tu et al., 2019; Zhao et al., 2024). Studies show that paeoniflorin (PF) promotes the translocation of Nrf2 into the nucleus, which significantly increases the expression of phase II detoxification enzymes like heme oxygenase-1 (HO-1) and NAD(P)H quinone oxidoreductase 1 (NQO1). This boost helps reduce oxidative damage in the lungs. Additionally, botanical extracts have been found to restore glutathione peroxidase (GPx) activity by epigenetically regulating the gene expression of HMGB1 and IL-22. Under oxidative stress conditions, the Keap1-Nrf2 protein complex undergoes structural changes and dissociates. However, persistent activation of Nrf2 could potentially lead to tumor development (Liu, Pi & Zhang, 2021; Sahu & Jain, 2025), underscoring the importance of carefully controlling both the strength and duration of pathway activation in therapeutic settings.

#### Synergistic regulatory mechanisms of MAPK and PI3K/Akt pathways

During the progression of AP-ALI, the MAPK and PI3K/Akt signaling pathways work together to regulate inflammatory responses and oxidative stress by interacting with multiple targets (Zhang et al., 2021). Experimental evidence shows that Hydrostatin-SN10 selectively inhibits IL-6-mediated JAK2/STAT3 signaling, which exhibits significant crosstalk with MAPK. This inhibition results in a notable decrease in the expression of pulmonary apoptosis-related proteins like Caspase-3 and BAX, while also increasing the production of the anti-apoptotic protein Bcl-2 (Yang et al., 2021). Additionally, baicalin specifically inhibits the TLR4/MyD88/TRIF signaling pathway, which is functionally linked to PI3K/Akt, effectively reducing reactive oxygen species (ROS) production (Ding et al., 2016) and substantially enhancing the activity of glutathione (GSH) and SOD, thereby improving pulmonary function parameters. These findings provide molecular evidence that targeted therapies aimed at the MAPK and PI3K/Akt pathways regulate multiple critical molecules involved in AP-ALI’s pathology. However, the exact molecular interactions between these pathways still need further investigation.

It is essential to recognize that intricate regulatory dynamics exist among these pathways, as illustrated by the opposing interactions between the transcription factors NF-κB and Nrf2, with MAPK signaling influencing both pathways in a bidirectional manner by modulating their activation and downstream effects. Furthermore, future research should utilize systems biology approaches to connect key nodes across different pathways, offering a detailed mechanistic foundation to inform the development of more effective treatments for AP-ALI.

### Clinical translation value of oxidative stress-related biomarkers

#### Dynamic alterations of oxidative stress biomarkers in AP-ALI

Throughout the progression of AP-ALI, significant changes occur in oxidative stress biomarkers (Soliman et al., 2025). Specifically, MDA levels, which are the end products of lipid peroxidation, are substantially elevated in animal models of AP-ALI. This increase indicates damage to biological membranes caused by oxidative stress (Fang et al., 2020). Pharmacological studies demonstrate that Rivastigmine (Riv) effectively lowers pulmonary MDA levels in AP models while enhancing the total antioxidant capacity (TAC) of lung tissue (Yehia Kamel et al., 2023). Key antioxidant defense components, such as SOD and GSH, often decrease in activity and levels in AP-ALI due to depletion (Luan et al., 2013). Mechanistic investigations reveal that Hydrostatin-SN10 significantly boosts pulmonary SOD activity and GSH levels, while reducing MDA concentrations by modulating the IL-6/JAK2/STAT3 signaling pathway, thereby decreasing oxidative damage. Additionally, the therapeutic effects of baicalin support the idea that modulating the TLR4/MyD88/TRIF pathway not only reduces MDA production but also restores the GSH-SOD antioxidant system (Ding et al., 2016). These findings highlight important links between changes in oxidative stress biomarkers and the development of AP-ALI.

#### Clinical application prospects of biomarkers

Oxidative stress biomarkers hold significant clinical importance for early detection and prognostic assessment of AP-ALI (Abu-Zidan, Bonham & Windsor, 2000; Tsai et al., 1998). Biomarkers such as MDA, SOD, and GSH can be evaluated using minimally invasive techniques like serum or bronchoalveolar lavage fluid (BALF) analysis, ensuring practical clinical use. Additionally, assessing oxidative stress biomarkers alongside pro-inflammatory cytokines, such as tumor necrosis factor-alpha (TNF-α) and interleukin-6 (IL-6), significantly enhances the accuracy of disease stratification (Li et al., 2021). Research on baicalin demonstrates its dual ability to lower MDA levels and inhibit NF-κB-mediated inflammation, providing valuable insights that improve prognostic evaluation. Moreover, modern omics research has identified that the upregulation of Nrf2/ARE downstream targets—including heme oxygenase-1 (HO-1) and NAD(P)H: quinone oxidoreductase 1 (NQO1)—is strongly associated with decreased oxidative stress (Joo Choi, Cheng & Shik Kim, 2014), suggesting these could serve as innovative prognostic biomarkers. Despite current clinical challenges like assay standardization, substantial preclinical data highlight the crucial role of oxidative stress biomarkers in early detection and targeted therapy for AP-ALI. Future multicenter clinical trials are necessary to validate these findings in a translational setting.

### Research advances in intervention strategies based on antioxidant mechanisms

#### Therapeutic value and limitations of classical antioxidants

N-acetylcysteine (NAC) and vitamin E, both recognized as key antioxidants, hold significant clinical importance (Polyak et al., 2018). NAC acts as a precursor to glutathione, offering protective benefits by enhancing the body’s natural antioxidant defenses. Research shows that in animal models, lung injury caused by acute pancreatitis is significantly reduced by NAC, which lowers markers of oxidative stress like MDA while increasing tissue levels of glutathione (Demols et al., 2000; Mokra, Porvaznik & Mokry, 2025). However, NAC’s clinical use is limited due to poor pharmacokinetic properties. Similarly, vitamin E, a potent lipid-soluble antioxidant, effectively prevents lipid peroxidation caused by free radicals. Nonetheless, its effectiveness decreases in complex disease conditions (Blaner, Shmarakov & Traber, 2021). Additionally, high doses of vitamin E may lead to adverse effects and have little impact on important inflammatory mediators such as TNF-α.

#### Experimental advances in innovative antioxidants

Selective antioxidants, such as molecular hydrogen and melatonin, have gained significant interest due to their unique mechanisms of action. Molecular hydrogen is noted for its highly selective ability to neutralize harmful reactive oxygen species like hydroxyl radicals while preserving cellular redox balance. In models of lung injury related to pancreatitis, hydrogen-rich solutions have been shown to substantially reduce tissue damage by modulating the NF-κB signaling pathway (Sun et al., 2021). Conversely, melatonin works through a dual mechanism: it not only activates the cellular defense transcription factor Nrf2 but also directly scavenges free radicals. Although molecular hydrogen has demonstrated notable antioxidant effectiveness in acute pancreatitis models, its pharmacological properties need optimization through innovative delivery systems (Zhao et al., 2024). Furthermore, the polypeptide Hydrostatin-SN10 provides unique therapeutic benefits by targeting multiple pathways. These findings suggest that pathway-specific antioxidant strategies could be a promising approach to improve therapeutic outcomes.

#### Current status of precision antioxidant therapy

The therapeutic strategy for AP-ALI has evolved from traditional broad-spectrum antioxidant supplementation towards precision medicine, focusing on specific pathways, advanced delivery systems, and cell-based therapies. Targeting key pathological pathways is a cornerstone of this approach. Ferroptosis, an iron-dependent form of regulated cell death driven by lipid peroxidation, plays a critical role in AP-ALI. The natural compound Wedelolactone (Wed) has been shown to upregulate glutathione peroxidase-4 (GPX4), a central inhibitor of ferroptosis, thereby reducing apoptosis in pancreatic acinar cells and mitigating both pancreatic and subsequent lung injury (Fan et al., 2021). To overcome the limitations of traditional antioxidants, such as poor bioavailability and lack of targeting, nanotechnology offers innovative solutions. Delivery systems based on natural polymers can significantly enhance drug efficacy (Obeng et al., 2025; Gu et al., 2024). However, key challenges remain, including potential off-target effects due to complex pathway crosstalk, insufficient long-term safety data for nanomaterials, and the difficulty in devising personalized treatment regimens. Future research should integrate gene-editing technologies with intelligent, responsive drug delivery platforms. Cell therapy, particularly using mesenchymal stem cells (MSCs), represents a promising systemic approach. For instance, hypoxia-conditioned MSCs have been demonstrated to attenuate acute lung injury by modulating the CXCL5/6-CXCR1 axis and enhancing regulatory T cell (Treg) recruitment and function (Yin et al., 2025). Through such multi-faceted immunomodulation, MSCs can trigger beneficial antioxidant cascades, offering a comprehensive therapeutic advantage over single-target agents. In summary, precision antioxidant therapy for AP-ALI is advancing through multi-level, multi-target strategies, with MSC-based treatments emerging as a key direction by leveraging networked biological effects.

## Research Perspectives

Contemporary research strongly indicates that oxidative stress plays a key role in the development of lung injury related to pancreatitis. Excessive ROS not only cause direct tissue damage but also worsen the disease by triggering complex inflammatory pathways. Although many preclinical studies show potential benefits of various antioxidant therapies, significant obstacles—such as pharmacokinetics, side effects, drug delivery efficiency, tissue targeting, and the need for personalized treatments-limit their progress into clinical practice (Seo, Katz & Snyder, 2025). There is a clear gap between experimental models and real-world clinical scenarios; animal studies often focus on single interventions, while human conditions usually involve multiple dysregulated processes affecting oxidative stress and inflammation, making translation difficult (Jiang et al., 2024). To overcome these issues, it is essential to develop combination therapies that leverage synergistic effects across multiple targets. Moreover, using precision medicine to identify patient subgroups likely to respond will greatly improve treatment outcomes. Both strategies are crucial for enhancing the effectiveness of therapies.

Progress in this area requires a collaborative effort between basic research and clinical practice. Standardized clinical trials are crucial for confirming the effectiveness of current treatments, while actively searching for new biomarkers for early diagnosis remains equally important. Achieving therapeutic progress depends on innovative collaborations across various fields—such as molecular biology, clinical medicine, and pharmacology. Ultimately, these efforts will lead to meaningful clinical benefits for patients affected by these conditions.

## Conclusions

This review offers valuable perspectives on the role of oxidative stress in acute pancreatitis induced lung injury. Excessive ROS production and dysfunction of the antioxidant system represent core pathological mechanisms. By activating signaling pathways such as NF-κB, Nrf2/ARE, and MAPK, this process promotes the release of inflammatory mediators, leading to disruption of the alveolar-capillary barrier, programmed cell death, and NETs formation, thereby exacerbating lung tissue damage. Oxidative stress biomarkers hold significant clinical value for early diagnosis and therapeutic efficacy assessment in AP-ALI, though their standardized detection and clinical application require further validation. while several limitations remain. A gap persists in the evidence chain linking fundamental mechanisms to clinical therapy, with inadequate characterization of the dynamic role of oxidative stress within complex organ interaction networks. Moreover, the review does not propose targeted therapeutic strategies with breakthrough potential based on precise mechanistic insights. Key challenges include the broad scope of research, mechanistic complexity, difficulties in clinical translation, and insufficient consideration of individual variability. Future studies should place greater emphasis on multifactorial interactions, develop treatment strategies with enhanced clinical applicability, and strengthen the accumulation of clinical data.

## Additional Information and Declarations

### Data Availability

The following information was supplied regarding data availability: This is a literature review.
